# Tuberculosis treatment adherence in the era of COVID-19

**DOI:** 10.21203/rs.3.rs-1777276/v1

**Published:** 2022-07-12

**Authors:** Christopher Lippincott, Allison Perry, Elizabeth Munk, Gina Maltas, Maunank Shah

**Affiliations:** Johns Hopkins University; New York University; Johns Hopkins University; Johns Hopkins University; Johns Hopkins University

**Keywords:** Video DOT, mHealth, tuberculosis, medication adherence, telemedicine

## Abstract

**BACKGROUND:**

In-person directly observed therapy (DOT) is commonly used for tuberculosis (TB) treatment monitoring in the US, with increasing usage of video-DOT (vDOT). We evaluated the impact of COVID-19 on TB treatment adherence, and utilization and effectiveness of vDOT.

**METHODS:**

We abstracted routinely collected data on individuals treated for TB disease in Baltimore, Maryland between April 2019 and April 2021. Our primary outcomes were to assess vDOT utilization and treatment adherence, defined as the proportion of prescribed doses (7 days/week) verified by observation (in-person versus video-DOT), comparing individuals in the pre- and post-COVID (April 2020) periods.

**RESULTS:**

Among 52 individuals with TB disease, 24 (46%) received treatment during the COVID-19 pandemic. vDOT utilization significantly increased post-COVID (18/24[75%]) compared to pre-COVID (12/28[43%], p=0.02). Overall, median verified adherence was similar pre- and post-COVID (65% versus 68%, respectively, p=0.96). Adherence was significantly higher overall when using vDOT (median 86% [IQR 70-98%]) compared to DOT (median 59% [IQR 55%-64%], p<0.01); this improved adherence with vDOT was evident in both the pre-COVID (median 98% vs 58%, p<0.01) and post-COVID period (median 80% vs 62%, p=0.01).

**CONCLUSION:**

vDOT utilization increased post-COVID and was more effective than in-person DOT at verifying ingestion of prescribed treatment.

## Background

Tuberculosis (TB) remains a public health priority in the United States (US).(1–3) TB treatment requires a significant investment of public health resources due to prolonged treatment courses with multi-drug regimens which are often complicated by drug toxicity.(2) These challenges can strain treatment adherence therein leading to suboptimal outcomes including treatment failure and drug-resistance.(4, 5) Adherence support interventions are beneficial for patients and promote successful completion of TB treatment.(1, 2, 6)

Directly observed therapy (DOT) is a strategy used to monitor TB treatment adherence.(2) While a Cochrane review concluded that DOT “did not provide a solution to poor adherence in TB treatment”(6), within the global community, DOT is a heterogenous strategy with context-dependent definitions, including community- or home-based DOT, which can be administered by trained lay providers, healthcare workers, or family members. World Health Organization (WHO) recommends DOT to support patients throughout treatment while not explicitly as an adherence support intervention.(7) In the US, DOT is under the jurisdiction of local health departments and typically involves a trained healthcare professional supervising a patient while ingesting TB therapy in the home or workplace.(2, 8) This strategy is recommended by the Centers for Disease Control and Prevention (CDC) as standard of care and has been a mainstay of TB case management and adherence monitoring for decades.(2) However, DOT has several limitations including inefficient use of human resources and inability to provide observed dosing on weekends; these challenges are exacerbated in the Coronavirus Disease 2019 (COVID-19) era with limitations on in-person close contact. Novel approaches are needed to improve upon this treatment adherence support strategy.

Video DOT (vDOT) is a modality where digital technology platforms (e.g. smartphone, tablet, computer) are used to verify the observation of a patient, either live or recorded, receiving their TB treatment.(9, 10) WHO have recommended vDOT be considered as an alternative to in-person DOT when technology is readily available to support this approach and CDC developed a toolkit to provide guidance for providers looking to utilize this strategy with their patients.(7, 9, 10) Since that time, vDOT has been increasingly utilized in the US, has shown to improve treatment adherence monitoring, in part by allowing for routine observation of weekend doses, and is broadly accepted by staff and patients alike.(11–13) Importantly, vDOT is economically feasible with significant cost-savings over the course of a 6 month treatment course.(11, 12)

In early 2020, the onset of the COVID-19 pandemic created a need to continue providing essential healthcare services while adopting social distancing practices to reduce the speed of viral transmission. This spurred a rapid transition towards telehealth clinical services beginning in March and April 2020 across health systems.(14) Since that time, the adoption of telehealth for outpatient clinical services has been massive with an estimated 63-fold increase in Medicare telehealth utilization during the COVID-19 pandemic.(15) However, the adoption of telehealth services for TB treatment adherence monitoring, specifically vDOT, during the COVID-19 pandemic has not been described. Many TB programs suffered worker shortages and health care service disruptions with negative consequences on TB outcomes.(16, 17) In Maryland, where this study was conducted, limitations on in-person contact instituted in April 2020 disrupted usual DOT services and in-person nurse case-management, and some health care workers were task-shifted to COVID response activities. Thus, we performed a retrospective cohort study to understand the impact of the COVID-19 pandemic on TB treatment adherence, and the utilization and effectiveness of vDOT before and during the COVID-19 pandemic at the Baltimore City Health Department (BCHD) TB Program.

## Methods

We conducted a retrospective observational cohort study of patients receiving routine TB treatment at the BCHD TB Program. The study protocol was approved by the Johns Hopkins Medicine Institutional Review Board.

### Study population

From April 2019 to April 2021, we enrolled patients receiving treatment for TB disease in Baltimore, Maryland. Routinely collected data was abstracted from eligible patients ≥ 18 years of age who were then classified into the pre-COVID period and post-COVID period. Individuals were classified as receiving treatment in the post-COVID period if they started therapy after April 2020, or received at least three months of therapy after April 2020 to capture those who may have initiated prior to April 2020 but received significant care during the post-COVID period. As part of routine clinical care at BCHD, all patients have treatment adherence monitored using DOT (in-person adherence verification through observation in person’s home or workplace 5 days per week excluding weekends and government holidays) or asynchronous vDOT (7 days per week) according to local protocols using a shared decision-making paradigm between patients and TB clinic providers. Patients and providers changed treatment monitoring strategies as needed based on individualized considerations; there were no restrictions or exclusions on vDOT usage based on prior adherence or treatment completion, sputum smear-status, or drug resistance.

### vDOT application usage

The vDOT system (emocha mobile health, Baltimore, MD) is comprised of a smartphone/tablet application (app) used by patients, and a web-based dashboard used by the TB clinic. The patient-side app reminds patients to take their medications on a schedule specified by the clinician. For non-English speaking patients, the app can deliver content in multiple languages customized to the patient. The app allows a secure/encrypted video recording to be taken as the patient ingests the prescribed medications, according to the patient’s schedule. Encrypted videos and data are transmitted to a secure cloud-based server within seconds and subsequently deleted from the patient’s phone, ensuring privacy. The videos can then be asynchronously viewed to verify medication adherence by TB clinic staff through the emocha web interface, typically on the next business day, where the clinician is prompted to make a determination as to adherence with DOT after watching each video.

### Statistical analysis

Primary outcomes were stratified by the pre- and post-COVID periods and included vDOT utilization and treatment adherence. vDOT utilization was defined as the proportion of patients who received any vDOT for treatment of TB disease. Treatment adherence was defined as the proportion of verified prescribed doses over 7 days per week. Standard descriptive statistics were used to characterize the study cohort. Chi-square test and two-sample t-tests were used to compare vDOT utilization and treatment adherence between DOT and vDOT overall and in the pre- and post-COVID periods. Multivariable logistic regression was used to understand the relationship between patient characteristics with utilization of vDOT and treatment adherence. All analyses were performed with Stata 16.

## Results

A total of 52 patients received treatment for active TB during the study period, of which 24 (46%) received therapy in the post-COVID period. Most patients were men (33, 63%), Black/African American (31, 60%), and born outside the US (29, 56%). Nearly all patients (51, 98%) were HIV-uninfected. Thirty patients were classified as having pulmonary TB (58%), 17 (33%) patients had extrapulmonary TB, and 5 (10%) patients had both. There was no difference in the distribution of pulmonary and extrapulmonary TB (p = 0.81) or AFB smear-status (p = 0.29) in the pre- and post-COVID periods. (Table 1)

### Reach of vDOT

TB treatment was monitored for all patients with vDOT, in-person DOT, or both. Overall vDOT was utilized for some portion of TB treatment monitoring for 58% (30/52) of patients while the remaining patients (42%, 22/52) received treatment monitoring with in-person DOT only. Among 30 individuals receiving vDOT, 14 (47%) received vDOT for their entire treatment course within the BCHD TB program. A significantly greater proportion of patients utilized vDOT during the post-COVID period (18/24, 75%), compared to the pre-COVID period (12/28, 43%, p = 0.02). (Table 2) Among individuals receiving some form of vDOT, the median proportion of all prescribed treatment monitored by vDOT was 75%, (IQR 53–92%); there was a trend towards a greater proportion of therapy monitored by vDOT comparing pre- and post-COVID periods (68% vs 73%, p = 0.58). In a multivariable analysis, vDOT utilization was most influenced by age (> 65, AOR 0.011 [0.0002-0.62], compared to age < 30) and the onset of the COVID-19 pandemic (AOR 16.6 [1.35–203] for post-COVID compared to pre-COVID, p = 0.03), and was not influenced by birth country (AOR 1.86 [0.18–19.2] for non-USB compared to USB), sex (AOR 1.5 [0.15–15.9]) for males compared to female), homelessness (AOR 4.65 [0.1–211]), employment (AOR 5.5 [0.7–43]), or smear-status (AOR 0.42[0.05–3.6]).

### Effectiveness of vDOT compared to in-person DOT

Median overall adherence (irrespective of monitoring strategy) was 66% (IQR 57–84%), and was similar in the pre- (65%, IQR 57–83%) and post-COVID (68%, IQR 57–84%) periods (p = 0.96). Overall, the proportion of prescribed doses (seven days/week for all patients) verified through observation was significantly higher when utilizing vDOT (median 86% [IQR 70%-98%]), compared to in-person DOT (median 59% [IQR 55%-64%], p < 0.01). These findings were driven by the high proportion of self-administered doses when using in-person DOT (median 38% [IQR 35–44%) compared to periods when patients were using vDOT (median 1% [IQR 0–7%]) (p < 0.01). (Table 2)

### Verified adherence in pre- and post-COVID periods

Verified adherence was higher when using vDOT in both the pre-COVID (median 98% vs 58%, p < 0.01) and post-COVID period (median 80% vs 62%, p = 0.01). When comparing the pre- and post-COVID period adherence for each treatment monitoring strategy, verified adherence was similar when using in-person DOT (median 58% vs 62%, p = 0.76) and declined slightly when using vDOT (median 98% vs 80%, p = 0.02). The latter difference was primarily attributable to an increase in the number of doses that were self-administered when using vDOT between the pre- and post-COVID periods (median 0% vs 6%, respectively, p = 0.03). Self-administered doses (i.e., weekends and holidays) when using in-person DOT remained high and stable across the pre-and post-COVID periods (median 38% vs 37%, respectively, p = 0.94). (Table 2) Overall, in a multivariable analysis, adherence was most influenced by receipt of vDOT and was 18% higher among those receiving vDOT compared to those not receiving vDOT; verified adherence was not associated with age, sex, birth country, homelessness, or study period.

## Discussion

The COVID-19 pandemic and associated lockdowns and staffing shifts led to significant disruption in healthcare delivery within TB programs including the Baltimore City Health Department, particularly related to traditional approaches to treatment verification using in-person DOT.(14, 15) With diminished ability to deliver in-person DOT, our study confirmed that there was a significant increase in vDOT utilization in the post-COVID period. Nonetheless, despite these COVID-related disruptions, this retrospective cohort study found that overall verified treatment adherence was not impacted by the COVID-19 pandemic. Moreover, we found that in both the pre-and post-COVID period, vDOT usage led to significantly higher verification of treatment compared to in-person DOT.

Overall, there have been shifting paradigms in TB treatment monitoring towards patient-centered approaches such as vDOT. Our study suggests that the COVID-19 pandemic may have accelerated these transitions. In Baltimore, prior to COVID-19, less than half of all patients were monitored using vDOT. With limitations on in-person contact during much of 2020 and 2021, we found that nearly three-quarters of patients were subsequently monitored using vDOT. Age greater than 65 was associated with decreased vDOT utilization, a finding previously noted in the vDOT literature, which may suggest that familiarity with smartphone/tablet technology may impact patient acceptance of this strategy.(18) Despite this vDOT transition, there was no decline in treatment adherence overall in the TB program; rather, individuals monitored with vDOT had a higher proportion of prescribed doses verified compared to in-person DOT in both the pre-COVID and post-COVID periods.

Our study therefore highlights particular limitations with in-person DOT in monitoring TB treatment, which is increasingly prescribed 7 days per week. We found that vDOT was more effective than DOT at verifying treatment adherence, and that less than two-thirds of prescribed therapy were able to be verified when using in-person DOT–reflecting logistical challenges with in-person verification during weekends and holidays. Consequently, we report that a large proportion of prescribed therapy is given with self-administration when using in-person DOT.

We also identified other challenges experienced by TB programs during the COVID-19 pandemic.(16, 17) Specifically, we found a modest decline in verified adherence when using vDOT, when comparing the pre-COVID (98%) to post-COVID (80%) period. This decline in vDOT verified adherence was driven by a significant increase in self-administered vDOT doses in the post-COVID period. The reasons for this finding are unclear, but were felt by the clinic staff to be attributable to individual level delays in transitioning and initiating vDOT due to reduced opportunities for in-person training on the vDOT system, or delays in troubleshooting any technical issues that may have been experienced by patients.

There are several limitations with this study. Since the study is an analysis of the real-world implementation of vDOT in a city health department, patients were not randomized to one modality or another. Although we report significant differences of verified adherence between vDOT and in-person DOT, the study was not designed to draw a definitive conclusion in this regard for all populations. Selection of patients for vDOT versus in-person DOT were made by clinic staff based on individualized considerations, and reflect routine clinical practices. While vDOT was the utilized by a significant majority of patients in the post-COVID period, additional qualitative assessments are needed to fully understand patient and provider perspectives associated with this transition from in-person DOT. Overall, vDOT had been available in the BCHD TB program since approximately 2016. Nonetheless, vDOT utilization increases appeared to be influenced by the COVID-19 pandemic. However, we cannot rule out alternative factors including increasing comfort and experience with the vDOT platform that may also have led to progressive increases in vDOT utilization over time.

## Conclusions

In conclusion, despite the significant disruption of healthcare activities during the COVID-19 pandemic, verified TB treatment adherence at the BCHD was sustained in the setting of increased vDOT utilization. Verified TB treatment was significantly higher with vDOT and adds another data point to the growing literature showing that vDOT improves upon in-person DOT for adherence monitoring and is readily accepted by patients.(11–13, 18, 19) It should be recognized that in-person DOT remains an important strategy for selected patients and can be coupled with other in-person adherence support interventions (such as nurse counseling and monitoring).(2, 7) In this study, we found that a minority of patients continued to use in-person DOT during the COVID-19 period due to individualized circumstances. Nonetheless, vDOT has intrinsic advantages for documentation of adherence in allowing treatment verification on weekends and holidays and allows dosing according to patient’s schedules, and may reduce stigma. Studies should continue to explore the implementation of this important strategy in real-world settings.

## Figures and Tables

**Table 1:** Patient Demographics (n = 52)

	Overall	Pre-COVID	Post-COVID	p
Age, median (IQR)	43 (30–57)	39 (29–54)	53 (37–60)	0.082
Sex, n (%)				0.477
Male	33 (63%)	19 (68%)	14 (58%)	
Female	19 (37%)	9 (32%)	10 (42%)	
Born outside US, n (%)				0.182
No	23 (44%)	10 (36%)	13 (54%)	
Yes	29 (56%)	18 (64%)	11 (46%)	
Ethnicity, n (%)				0.025
Not hispanic	31 (60%)	12 (43%)	19 (79%)	
Hispanic	10 (19%)	7 (25%)	3 (13%)	
Unknown/not reported	11 (21%)	9 (32%)	2 (8%)	
Race, n (%)				0.364
Asian	9 (17%)	4 (14%)	5 (21%)	
Black/African American	31 (60%)	15 (54%)	16 (67%)	
White	6 (12%)	4 (14%)	2 (8%)	
Unknown/not reported	6 (12%)	5 (18%)	1 (4%)	
Experiencing homelessness, n (%)				0.573
No	45 (87%)	22 (79%)	23 (96%)	
Yes	5 (10%)	4 (14%)	1 (4%)	
Unknown/not reported	2 (4%)	2 (7%)	0 (0%)	
HIV-infected, n (%)				0.275
No	51 (98%)	28 (100%)	23 (96%)	
Yes	1 (2%)	0 (0%)	1 (4%)	
Tuberculosis classification, n (%)				0.809
Pulmonary	30 (58%)	15 (54%)	15 (63%)	
Extrapulmonary	17 (33%)	10 (36%)	7 (29%)	
Both	5 (10%)	3 (11%)	2 (8%)	
AFB smear, n (%)				0.290
Smear-negative	29 (56%)	18 (64%)	11 (46%)	
Smear-positive	22 (42%)	9 (32%)	13 (54%)	
Unknown/not reported	1 (2%)	1 (4%)	0 (0%)	

**Table 2:** Tuberculosis Treatment Adherence Before and During the COVID-19 Pandemic

	Overall	Pre-COVID	Post-COVID	p
vDOT utilization, n (%)	30/52 (58%)	12/28 (43%)	18/24 (75%)	0.019
Overall adherence, median (IQR)	66% (57–84%)	65% (57–83%)	68% (57–84%)	0.959
Verified Adherence by modality, median (IQR)				
vDOT adherence^1^	86% (70–98%)	98% (78–99%)	80% (60–93%)	0.022
DOT adherence^1^	59% (55–64%)	58% (53–61%)	62% (55–66%)	0.759
Missed Doses by modality, median (IQR)				
vDOT missed^2^	5% (0–16%)	2% (0–17%)	8% (0–16%)	0.555
DOT missed^2^	1% (0–5%)	2% (0–7%)	0% (0–2%)	0.276
Self-Administered Doses by modality, median (IQR)				
vDOT self-administered^3^	1% (0–7%)	0 (0–0%)	6% (1–16%)	0.026
DOT self-administered^3^	38% (35–44%)	38% (35–44%)	37% (33–44%)	0.941

1. Adherence was significantly higher when comparing vDOT to DOT overall (p < 0.001), during the pre-COVID period (p < 0.001), and during the post-COVID period (p = 0.012)

2. Missed doses were significantly higher when comparing vDOT to DOT overall (p = 0.008) and during the post-COVID period (p = 0.006). Missed doses were similar when comparing vDOT to DOT in the pre-COVID period (p = 0.321)

3. Self-administered doses were significantly lower when comparing vDOT to DOT overall (p < 0.001), during the pre-COVID period (p < 0.001), and during the post-COVID period (p < 0.001)

## Data Availability

The datasets generated and/or analyzed during the current study are not publicly available in the absence of a Data Transfer Agreement due to data sharing policies from the Baltimore City Health Department in conjunction with data security policies from Johns Hopkins University, but are available from the corresponding author on reasonable request.

## Abbreviations

AOR: adjusted odds ratio
App: application
BCHD: Baltimore City Health Department
CDC: Centers for Disease Control and Prevention
COVID-19: Coronavirus Disease 2019
DOT: directly observed therapy
IQR: interquartile ratio
TB: tuberculosis
US: United States
vDOT: video DOT
WHO: World Health Organization
